# Primary health care response to noncommunicable diseases: an assessment of Wellness Clinics in Ghana

**DOI:** 10.1186/s12913-024-11264-w

**Published:** 2024-07-10

**Authors:** Mawuli Komla Kushitor, Judith William, Deborah Esaa Larbi-Sarpong, Mary Akua Ampomah, Prince Owusu Adoma, Kennedy T. C. Brightson, Sandra Boatemaa Kushitor

**Affiliations:** 1 The Department of Health Policy, Planning and Management (HPPM), University of Health and Allied Sciences (UHAS), PMB 31, Ho, Ghana; 2 Deparment of Community Health, Ensign Global College, Kpong, Ghana; 3 The Department of Family and Community Health, University of Health and Allied Sciences (UHAS), Ho, Ghana; 4 Department of Health Administration and Education, University of Education, Winneba, C/R Ghana; 5 Shai-Osudoku District Hospital, Ghana Health Services, Dodowa, Ghana; 6 Deparment of Food Science and Centre for Sustainability Transitions, Stellenbosch University, Stellenbosch, South Africa

**Keywords:** Noncommunicable diseases, Primary health care, Wellness clinics, Policy coherence, Ghana

## Abstract

**Background:**

Globally, there is a significant unmet need for the rapidly growing burden of Non-Communicable Diseases (NCDs). Ghana has adopted and implemented Wellness Clinics (WC) nationwide to respond to the rising burden of NCDs. Regrettably, very little is known about WCs, including their structure and the services they offer. This study explores the concept of WC, their structure, position within the hospital environment, and services from the perspectives of healthcare providers and clients.

**Methods:**

An exploratory qualitative study was conducted with health professionals (*n* = 12) and clients (*n* = 26) of Wellness Clinics in two district hospitals and one regional hospital in a deprived region of Ghana where NCDs are rising. Using the WHO-PEN approach, an interview guide was purposely designed for this study. The data were analysed thematically using Atlas.ti.

**Results:**

All three Wellness Clinics were sub-units under the outpatient department. The WC was created by the facilities to respond to the increase in NCDs and to meet annual performance review requirements. The Wellness Clinics provided NCD diagnosis, counselling, and treatment services to approximately 300 clients per week at the facility level. Only one of the WCs provided NCD prevention services at the community level. Integrated NCD care was also provided at the WC, despite the health system and individual-level challenges reported by the health workers and clients.

**Conclusion:**

The implementation of the Wellness Clinic demonstrates the government’s commitment to addressing the increasing burden of NCDs in Ghana through the primary health system. To maximise the impact of the wellness clinics, we recommend developing best practices, providing logistics, and addressing health insurance challenges.

## Background

Globally, chronic non-communicable diseases (NCDs) are the leading causes of death [1]. Most of these deaths are due to cardiovascular diseases, cancer, chronic respiratory diseases, and diabetes. Low- and middle-income countries (LMIC), including countries in Sub-Saharan Africa, have a disproportionate NCD morbidity and mortality burden [1]. Despite the increase in diabetes and dyslipidaemia, population-based preventive programs and interventions are inadequate, and healthcare for these conditions remains poor [2]. In 2012, the World Health Assembly (WHA) Resolution on preventing and controlling NCDs recognised the need for global surveillance of the common modifiable risk factors of NCDs and recommended an integrated approach to health provision [3]. Following the WHO call was the Lancet Commission on Diabetes publication in 2020 on using data to improve patient care and diabetes prevention [4].

In 1978, Primary Health Care (PHC) was declared as the cornerstone of health systems through the Alma-Ata declaration [5, 6]. Strengthening the PHC system is seen as the most inclusive and effective approach to providing health services to all [5]. Regarding NCDs, PHC systems support NCD control through health promotion and improving the quality of life of individuals living with NCDs by reducing complications and premature deaths, especially in low- and middle-income countries. In 2022, the WHO designed the Package of Essential Non-communicable (PEN) disease interventions for PHC to strengthen early detection and timely treatment [7]. Table 1 contains the guidelines for this package. Most importantly, the interventions are cost-effective and can be delivered in resource-poor settings.

**Table 1:** WHO-PEN toolbox with the enabling actions

1. Explore viable health financing mechanisms and innovative economic tools supported by evidence. 2. Scale up early detection and coverage, prioritizing cost-effective, high-impact interventions. 3. Train the health workforce and strengthen the capacity of health systems, particularly at the primary care level, to address the prevention and control of noncommunicable diseases. 4. Improve the availability of the affordable basic technologies and essential medicines, including generics, required to treat major noncommunicable diseases, in both public and private facilities. 5. Strengthen and orient health systems to address noncommunicable diseases and risk factors through people-Clinics health care and universal health coverage. 6. Develop and implement a palliative care policy, including access to opioid analgesics for pain relief, together with palliative care training for health workers. 7. Expand the use of digital technologies to increase health service access and efficacy for NCD prevention, and to reduce the costs in health care delivery.	

Source: WHO-PEN, 2020

Currently, NCDs account for the majority of all-cause mortality in Ghana, with major NCDs such as heart diseases, strokes, diabetes, cancers and respiratory diseases dominating outpatient departments [8]. In Accra, about one-fourth of out-patient clients had an NCD [9] and about 5% of homeless residents had diabetes [10]. Despite the rising burden of NCD morbidity and mortality, awareness, treatment, and control of NCDs have remained critically low [11]. The high morbidity and mortality from NCDs directly result from the inadequate NCD response [12, 13]. Like in many African countries, Ghana’s health system is historically structured to treat infectious diseases, which are often characterized by one-time treatments [14]. Therefore, continuous management and integration of different specializations required for NCD management are not built into health system processes in a way that delivers adequate NCD care systematically [15].

In recognition of this structural challenge, the Ministry of Health and the Ghana Health Service has initiated some national interventions to improve the health system’s response to NCDs. These interventions were based on the National Health Policy, the National Policy Non-Communicable Diseases and the National Health Promotion Strategy 2022–2026 [16, 17]. These policies informed the creation of the Wellness Clinics (WC) by the Ghana Health Service. These clinics were created to ensure that Ghanaians maintain healthy lives by providing a space/desk for wellness checks from health professionals with basic logistics. The clinics were aimed at counselling, screening and referring ‘well’ Ghanaians for NCD services. In 2020, the Ghana News Agency reported the opening of the first Wellness Clinics in two regional hospitals. According to the report, ‘Wellness Clinics’ at all primary healthcare facilities will give unfettered access to people to walk in without demand for any hospital attendance card, health insurance or any demand whatsoever to check their vital health indicators for free [18].

However, standard operating procedures for establishing and running the centers were not provided after the launch. Informal conversations with district health directors and health administrators have demonstrated a limited understanding of how to structure the WC and integrate it into routine care. Therefore, the WC, similar to other NCD interventions, has suffered from the difficulty of integrating and implementing interventions at the sub-national level [19]. This study explores WC within the broader frame of the WHO-PEN strategy, especially packages 2, 3 and 5, which promotes scaling up of detection, training NCD competent workforce and strengthening health systems. This study explores the concept of WC, its structure, position within the hospital environment, and services from the perspectives of healthcare providers and clients. This study contributes to providing conceptual clarity, functional identity and increased understanding of wellness clinics in Ghana.

### Primary health care context in Ghana

In Ghana, health care is provided on three levels: primary, secondary and tertiary [20–22]. The primary level comprises Community-Based Health Planning and Services (CHPS), Health Centers and District Hospitals. CHPS remains Ghana’s flagship program for providing the Universal Health Coverage (UHC). These are small community clinics built to deliver health care within the context of the community. At the secondary level, health care is delivered through regional hospitals [23]. Tertiary hospitals sit at the apex of this very complex structure. Tertiary hospitals are designed to provide highly specialised care that is over and beyond what is provided by the primary level and the secondary level. Besides this categorisation, the Ghana Health Service (GHS), which oversees health care delivery in Ghana, is administratively organised at national, regional, and district levels. The national administration, headquartered in Accra, the capital, oversees the regional administrations. The regional health directorates govern the district administration. Ideally, the district directorate is the point through which primary health care is delivered. The District Health Management Teams (DHMT) organize and manage the health facilities within the districts. They oversee the activities of all healthcare services within each district. WC can be found at some health centers, district, regional and tertiary hospitals.

In the 2013–2017 National NCD Policy and Strategy, the Ministry of Health stated that “The WHO Package of Essential NCD Interventions (WHO-PEN) will be used to provide NCDs prevention and control services at the primary health care level” [24]. The Ministry maintained this position in the revised NCD policy in 2022 [16]. At the national level, a 5-year project using the PEN approach was launched in 2021 [25]. Despite the presence of various programs, the WC in Ghana are not well-defined. There are no operational manuals or guidelines for staffing or service delivery.

## Materials and methods

### Study design and setting

An exploratory qualitative study design was adopted to understand the concept of Wellness Clinics in one regional hospital and two district hospitals in a deprived region of Ghana. Similar to the nation, the burden of NCDs is rising in this region, and access to care is limited. Letters were sent to all facilities (*n* = 18) in the region that were operating wellness clinics in 2021. Only three facilities responded due to fear of being identified after several follow-up visits. These facilities included a regional hospital and two district-level hospitals (Table 2). Health Facility 1 was established in the 1950s as a community clinic with about 30 to 60 beds. In 2000, the bed capacity was increased to about 150 beds. Facility 2 was established after 1950. Currently, it is a 140-bed capacity hospital facility. Facility 3 was established in the 1920s-30s. It is a more than 100-bed capacity hospital. At each facility, all the health workers delivering services at the Wellness Clinic were invited to participate in the interview. The participants were selected at the facility on their appointment days. All those who were waiting to be attended to were invited to participate in the study. Only those who consented were interviewed.

**Table 2:** Summary description of wellness clinic interviewed

Facility level	Facility 1	Facility 2	Facility 3	GHS/MoH
	Regional hospital	District hospital	District hospital	
Number of staff	4	6	11	
Date facility established	1950s	After 1950	1920s-1930s	
Cadre of staff interviewed	Dietician (*n* = 1) Nurses (*n* = 1)	Doctor (*n* = 1) Nurses (*n* = 2)	Nurses (*n* = 2) Clinical psychologist (*n* = 1) Nutritionist (*n* = 1)	High-level administrators (*n* = 3) Officer of MoH (*n* = 1)
Conditions commonly attended to at the Wellness Clinic	Hypertension Diabetes Sickle cell	Hypertension Diabetes HIV/AIDS	HIV/AIDS Hypertension Diabetes	Not applicable
Number interviewed	Staff (*n* = 2) Clients (*n* = 11)	Staff (*n* = 3) Clients (*n* = 8)	Staff (*n* = 4) Clients (*n* = 7)	

### Description of facilities, healthcare workers and participants

Twelve healthcare providers were interviewed. Many of the health workers were female (*n* = 8). They were all below the age of 50. Seven of the health workers respondents were married, and five were single. There were eight nurses, one dietician, a clinical psychologist, a nutritionist, and a medical doctor. The senior medical officers were a medical superintendent of the district hospital, three high-level administrators, and an officer from the Ministry of Health.

The patients comprised 26 participants, many of whom were females (*n* = 19), and a few were male (*n* = 7). Twenty-two (22) of the patients were above the age of fifty. Ten (10) were married, five (5) were single. Ten were widowed, and one was divorced. Almost all of the respondents (*n* = 19) had some form of education, and seven (7) had no formal education.

### Data collection

In-depth interviews were conducted with staff (*n* = 12) and clients (*n* = 26) of the WC. Using the WHO PEN approach, an interview guide was purposely designed for this study. The interview guide explored the activities of the WC and how it has been structured in each facility to deliver NCD care. The client interviews explored the services they had received from the WC, care continuity and integration. The interviews were conducted in English and the local language preference of the respondent. Five allied health professionals who understood Ghana’s health system conducted the interviews and analysis.

### Data analysis

**Fig. 1:**
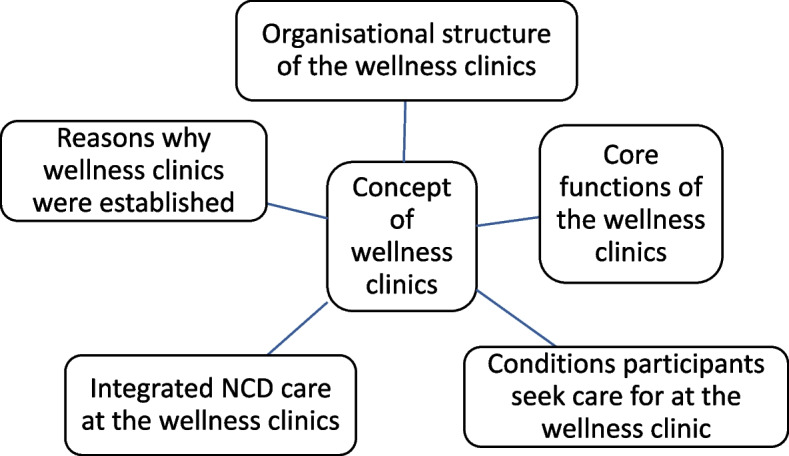
The wellness clinics and its core functions in Ghana

The data collected were transcribed and checked thoroughly for any errors. A thematic approach was adopted in analysing the transcribed data [26]. Transcripts were read thoroughly to ensure familiarity with the data. The first six transcripts (three for health workers and three for participants) were independently coded by three authors (MKK, JW and DELS) to generate the initial codes. Disagreements between the coders were discussed at a team meeting. A coding frame was developed based on these transcripts and applied to the remaining transcripts (Fig. 1). The fractured approach was used, and every transcript line was labelled with its own code when applicable. This method provides a vivid, multi-textured picture of the data and ensures the credibility and dependability of the research findings.

After the initial coding, SBK and JW compared the data associated with a code and modified it when necessary to ensure constant comparison. The codes were then collated based on their shared patterns to form sub-themes and central themes. Where necessary, themes were collapsed or combined to arrive at a defined pattern of shared meaning with a central idea. Transferability was achieved in this study by presenting the findings from the initial analysis to the medical superintendent in two facilities in the Greater Accra Region and an officer from the policy, planning, monitoring and evaluation unit of the Ministry of Health. They confirmed that the results are applicable to their setting. The analysis was aligned with the WHO-PEN enabling actions. The coding was done using ATLAS-ti version 7.5.7 by ATLAS. ti GmbH, Berlin.

## Results

The results are presented in three sections 1) the concept of the wellness clinic, 2) the perceptions of the impact of the wellness clinic and 3) challenges affecting the continuity of care at the wellness clinic. In each section of the results, dominant themes are presented first, followed by minor themes. Quotes from the transcripts and figures are included for illustrations.

### The concept of the wellness clinics in Ghana

Five major themes were identified to describe a wellness clinic and its core functions (Fig. 1).

### Organizational structure of the wellness clinics

This section presents information about the department of the WCs, their staff strength and physical location within the facilities (Table 3). All three WCs were sub-units under the outpatient department (OPD). The OPD was an open area connected to several consulting rooms of physicians, dieticians, clinical psychologists, nutritionists, and others. Nurses assigned to the WC took advantage of the physical structure of the hospital and, through personal contacts, got the services of key professionals to support the WC. The physical architectural design of the hospital OPD space allowed the WC to benefit from all the allied professionals. The disadvantage, however, was that the WC had to compete with other care clinics for the same limited resources. The clinics report to the public health directorate. The idea of setting the WC under the public health directorate of the hospitals was to facilitate continued management and support.

**Table 3:** Organisation structure of the wellness clinics and their outcomes

Variable	Description	Outcomes
Hospital organogram	Wellness clinic is a unit under the outpatient department	- Mainly facility-based screening and treatment services
	Wellness clinic reports to the public health directorate	- Community non-communicable disease prevention
Core staff	Nurses	- Nurses are the first cadre of healthcare professional clients meet, and their vitals are examined
	Doctor	- Clients seek all consultation services at the WC - WC without doctors, refer their clients to the general OPD
	Allied health professionals such as public health nutritionist, disease control officer, a health information person, health promotion officer	- Clients receive integrated care at facilities with allied health professionals at the WC
Physical capacity	No office space	- Clients go through the regular outpatient department and experience delays in accessing care
	Consulting room in the OPD	- The wellness clinics had to compete with other hospital departments for the same resources at the OPD

### Reasons why wellness clinics were established

Most participants stated that WC were created in response to the high burden of NCDs. They felt the need to appropriately and sufficiently respond to NCDs in their localities. WC was thought to be the means to address all the complexities of managing NCDs. All the facilities had established clinic days to prioritise diseases such as diabetes and hypertension, where the sheer number of people requiring regular treatment has necessitated special clinic days. The disease-specific clinics were sometimes referred to as wellness clinics by the patients.

To reduce the rise in NCDs:

*We realised the non-communicable diseases were increasing, so based on the data we had in the facility to analyse, diabetes and hypertension were rising, so community outreaches were done, and then the establishment took off. It’s because there was an increase in non-communicable diseases. (Facility 2)*

For specialised care:

*It is because of the number of hypertension cases, diabetes cases, sickle cell and asthma cases reported at the OPD, and they deem it necessary to gather them to give them health education. It is not just the medication; it is also about diet management, exercise, etc. These are the main reasons why the clinic was established. (Facility 3)*

According to a health provider in Facility 3, WC was established to fulfil health facilities’ annual peer review requirements. Comments from one facility illustrate this point.

*Performance. The regional officers would come and assess your performance with some facility indicators. This wellness clinic was one of the indicators that they needed to assess. (Facility 3)*

### Core functions of the wellness clinics

Health providers reported that each facility was allowed to develop its core functions as they deemed fit and to provide services based on the needs of their clients. This was succinctly captured in a quote by a respondent from Facility 3:

*It depends on the facility or hospital to determine what the Wellness Clinic does. For instance, they can focus on only maternal care, hypertension, monitoring children under five nutrition, etc., but we decided to work on all the conditions they present. (Facility 3)*

**Fig. 2:**
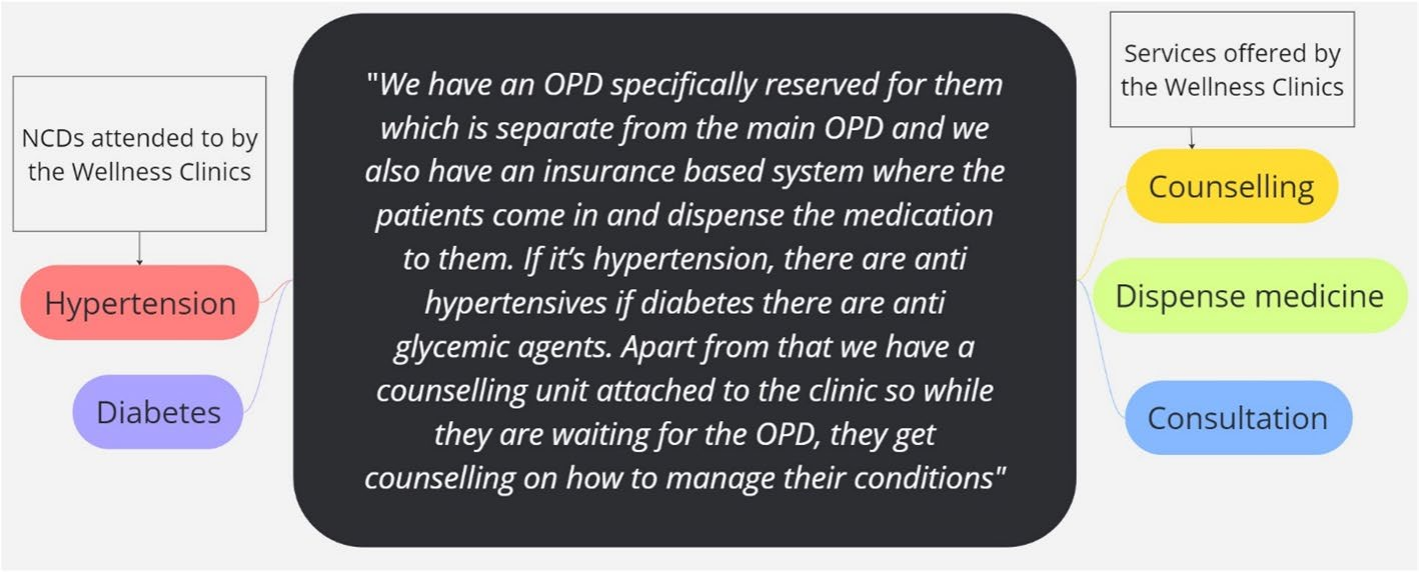
Clinical services provided by the wellness clinics

In practice, all three facilities provided NCD care and prevention services (Fig. 2). Facility 3 conceptualised WC as an arm of the hospital and provided early detection of NCDs. Wellness Clinics were seen as an entry point for further referrals to specific NCD clinics based on initial clinical suspicions. The WC was focused on hospital-based screening services. Individuals in the hospital are welcome to walk in for free medical advice and NCD testing. Blood pressure measurements were free, while other tests requiring consumables, such as blood glucose levels, were taken at a cost. Nurses and health providers attempted to take advantage of the flow of people into the OPD to screen for diseases such as hypertension and diabetes.

In Facility 1, the core function of the WC was providing integrated NCD care. The WC consolidated all NCD-related activities into the unit and provided disease-specific clinic days through the unit. The unit registered patients newly diagnosed with NCDs and linked them to care. It coordinated NCD referrals, regular monitoring, and patient follow-ups.

In Facility 2, the WC was designed to operate under the public health unit of the hospital. Like in Facility-3, they helped identify, detect, and recruit NCD patients. Once a condition was detected, clients were referred to the general OPD for mainstream medical attention.

Community services provided by the wellness clinics included health promotion. Facility 2 had a full complement of public health officers in the WC to engage patients outside of the hospital. They had a public health nutritionist, a disease control officer, and health information person and a health promotion officer to drive NCD care in the community. They launched all that NCD-related activity from the WC.

### Conditions participants seek care for at the Wellness Clinics

Hypertension (*n* = 9) and diabetes (*n* = 7) dominated the activities of WC in all three facilities (Fig. 2). ART (*n* = 1), Asthma (*n* = 1), HIV/AIDS (*n* = 1), hyperlipidaemia (*n* = 1), cardiac issues (*n* = 1) and stroke were other health conditions mentioned by some of the respondents. Two facilities, however, mentioned sickle cell as an essential focus of their WC.

Some responses were;

*We manage diabetes, hypertension, and sickle cells disease; I learned now that they add asthmatic patients, so last week, we treated some people. (Facility-1)*

### Integrated NCD care at the wellness clinics

To provide integrated NCD care, we assess how interprofessional health teams collaborate at the wellness clinics. In Facility 1, NCD patients were referred to the WC for continued support after detection and diagnosis. The WC was structured as an in-hospital support system to facilitate NCD care. The WC attempted to coordinate comprehensive and continuous NCD care and management. This support included continuous dietary counselling through group and individual sessions. The WC also arranged psychological counselling for sickle cell patients and physiotherapy services for those who needed such. They arranged personal nutrition and diet therapy when it was requested or when it was deemed necessary.

*Clients start from the OPD, but we have a computer over here just for them because we don’t want them to be stressed out, so they start with activating their cards, that’s with diabetes and hypertension before they come to us. We check their BP before giving them their cards; from the nurses, the patient goes to see the doctor; if the person must go to the lab, they are taken to the lab. But if the lab is not needed immediately, we sometimes write for the person to return for the lab tests on their next review date. Then, to the pharmacy. Sometimes, the nutritionists come here to give them a talk, or we send the patient to the nutritionist for the patient to be educated on the diet. We also have people going to the physiotherapy unit, especially patients with stroke. So, we all work together. Sometimes, if a pharmacist doesn’t understand a prescription, they come to us to explain it to them. So, we all work together. (Facility 1)*

In Facility 2, a newly identified NCD client would first be referred to the main hospital OPD for general assessment. At the OPD, clients are provided with all the required treatments and referrals. After receiving definitive NCD treatment from the OPD, patients were referred to the WC for continued support and management. The WC now take charge of the patient and supports the patient with regular dietary counselling and other affiliate support necessary for optimal NCD management. Facility 2 did not mention the services of a dietician, but they had a public health nutritionist who provided dietary counselling and nutritional therapy when deemed necessary.

In Facility 3, the WC had limited functionality by their own account. They had hoped to be able to have and to do more for NCD management. They needed a physical space in the facility and could not provide continual support for NCD management in the way they had wanted. They felt hampered by the physical space limitation, which prevented them from providing services to address the rising alcohol-related diseases such as liver conditions, hypertension and even diabetes. Most of the frustrations of the WC were related to continual management of NCDs after initial diagnoses and treatment.

### Health workers’ perception of the impact of WC on NCD management

Health workers feel that WC are performing well in terms of service delivery. They supported this claim with the number of people coming to the WC. All three facilities mentioned the growing numbers of people living with NCDs patronising their facility as an essential indicator of the WC’ impact in the districts. In all three facilities, daily attendance was more than fifty clients. According to the in-charge at Facility 1, more than 300 clients had self-registered with the WC for continual support with NCD management:

*In a day, we can attend to about 50 to 70 clients. They come more at the beginning of every month because they say there is money for transportation at that time. So, I was first told they attend to about 100 clients a day. (Facility 1)*

### Challenges affecting continuity of care at Wellness Clinics

**Fig. 3:**
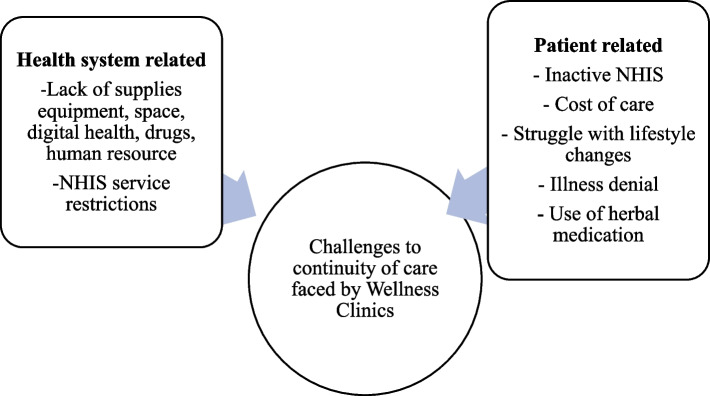
Breakdown of challenges affecting continuity of care at Wellness Clinics

The WC aimed to provide continued healthcare for their clients. However, health system and patient-related factors limited this goal (Fig. 3). The predominant challenge was the need for more supplies such as drugs, space, digital health services and human resources. Most registered clients wanted to secure some regular out-of-hospital communication with staff. That wasn’t easy to attain because hospital staff could not respond to most calls. The non-availability of drugs was the most critical complaint of health workers. Clients having to buy medication continuously was a significant drawback for WC. In addition to medicines, essential equipment (*n* = 8) and financial resources (*n* = 6) were noted as having important limitations to the WC services.

*For instance, with hypertension, there are those who have insurance and those who are finished and the person will not get the medication from our dispensary. We write for them to go and then get it in town. And that is another challenge, too, because the patient usually doesn’t have money to get the medication. Not all drugs are on the NHIS. Even with those on it, you may sometimes come, and it is finished. Ideally, all the medications are on the NHIS, but not for all conditions. For instance, with diabetes, we have a few medicines that are not on it, and they are a little bit expensive. (Facility 1)*

Health providers were deeply concerned about spacing and privacy (*n* = 3). In many places, there was no room for private consultations. Further, getting medical doctors and other essential services to support NCD consultations was sometimes challenging. WC were caught up in this complexity of dealing with chronic care in mostly deprived areas.

*For human resources now it’s a bit better, and also space, because of that we have to share the office, so privacy is not there. We need more space. Confidentiality for a client is compromised. Shortage of medication, sometimes even a basic drug you won’t get from the pharmacy. (Facility 2)*

Further, participants recognised the limited application of national health insurance for many people living with diverse NCDs (*n* = 6). Although some services are accessible at the WC, those who need drugs, pharmaceutical services, and laboratory services must pay for them. The services are not covered by insurance. Most clients could not continue pharmacotherapy because some medications were not covered or available in the hospital pharmacy.

*Sometimes the medicine is expensive, and one-time, I came, and they asked me to go to the lab, but it was costly, so I told him I couldn’t do it, and I didn’t do it. (Participant 1)*

Health workers also complained about expensive laboratory services that are unavailable to most older patients who are deprived through the NHIS (*n* = 3).

Above all, WC continuously struggled with illness denial and the client’s inability to accept the prognosis of a long-term condition. They seemed unwilling to accept the concept of long-term management and continuous and sometimes indefinite engagement with the healthcare system.

*Some are about compliance after taking the medication. Education is also a factor. Some think that after taking the medicine for one year, I still have this condition; why should I continue? It won’t do anything. So, they throw the medicines away and turn to herbal medications. (Facility 3)*

*Some people are given days to show up but don’t come for their medications on time, they stay at home, and then they get complications before they are brought in, in a lousy state at which you would now have to do other services, some come in with strokes, and it’s now an emergency. So, in my experience, it is an excellent initiative to have a special clinic set up for them (Facility 3)*

Other difficulties associated with patients were making significant changes to their lifestyle in diet management, alcohol consumption and regular physical activity.

## Discussion

In response to the growing burden of NCDs, the Ministry of Health and Ghana Health Service adopted Wellness Clinics as a primary health care initiative. This study explored WC in primary health facilities in a region of Ghana. Health providers in charge of WC and allied professionals who are integral in the structure and functioning of WC provided perspectives and shared their experiences. Although health facilities acknowledged that the idea was initiated and advanced by the Ghana Health Service (GHS), they did not have any documentation, training or a policy document to model the WC on. Therefore, all three WC recruited had a different understanding of the concept. The differences reflected how WC was positioned, structured and functioned. Each facility, therefore, built its concept, usually from its needs and resources. We propose two potential explanations.

Firstly, senior health administrators’ reflections on the concept and structure of the WC indicate that the WC was primarily created as an arm of the hospital system to initiate and sustain NCD detection. The officers were concerned that the WC providing the diabetes and hypertension clinics had deviated from their original orientation. They mentioned that this was due to the need for more documentation on the structure of these clinics. Senior hospital administrators noted that they only received a memo to start the WC but had limited information on specific strategies to set up and run them. Several attempts by this research team to retrieve the memo from the administrators and GHS availed nothing. The memo could not be found. The silence on this policy also reflects national NCD policy; WC is mentioned in cursory and referenced twice in the entire document. The organogram for NCD prevention does not include the Wellness Clinics [16].

Secondly, challenges with policy coherence can also explain the findings. The findings of this study indicated that there was no integration and alignment across facilities. Also, the facilities adopted siloed operations, and even though they were located in the same region, none of the respondents’ mentioned collaboration between the facilities. There is a need for collaboration as it can improve the sharing of innovations, best practices, and insights, which helps healthcare professionals learn and grow continuously. Additionally, combining resources and experiences can assist in lowering the facility’s overall operating expenses. The challenge of policy incoherence reported by the Wellness Clinics is similar to other programs implemented by the Ghana Health Service and broader in the policy arena [27]. Most regional and district-level officers are unaware or have inadequate knowledge of new programs in maternal health [28] and HIV/AIDS transmission [29]. These findings suggest a structural challenge of policy coordination at sub-national levels.

India has quite a robust Health and Wellness clinic focused on early screening and detection activities [30]. India has transformed its Peripheral Health Clinics (PHC) into Health and Wellness Clinics (HWC). It has substantially increased the content of treatment of the PHC to include more options, particularly for NCDs. In addition to the early detection, they were designed to provide significant clinical services at the PHC [30, 31]. Compared to the HWC in India, Ghana’s WC are limited in their core functions. Ghana’s WC were only found in district-level facilities, which restricts their accessibility to a wider population who primarily seek care from the CHPS and Health Clinics. Secondly, a significant part of the population will be missed since people who feel well do not attend clinics, even though such individuals may have NCDs and are unaware [32, 33]. The opportunity to use the public health unit as part of the wellness clinics should be considered so that community-based prevention and screening initiatives can be organized, as shown in Table 2.

To the best of our knowledge, this study is the first to generate information on WC in Ghana. It represents the inaugural effort to produce data on Wellness Clinics (WC) in Ghana, thus significantly contributing to the existing body of literature. It offers a comprehensive examination of the management practices employed in selected region of Ghana’s wellness clinic initiative. Yet, the study has limitations. In particular, the sample was based in only one region of Ghana. Considering the diversity of the conceptualisation of WC in the study area, it may be possible that WC may be conceptualised differently in others regions. Nevertheless, the study’s findings align with the media reports on the functions of the WC [18, 34]. According to the WHO-PEN, the concept of WC provided by this study can support the delivery of NCD services. Firstly, the findings can inform other primary health facilities that need clarity in designing WC. There was no mention of any specialised training regarding the operations of WC. It has become imperative that the 2022 NCD policy achieves its goal of increasing the percentage of district, Health Centres and CHPS front-line staff trained to carry out NCD screening and management services [16]. Furthermore, the policy documents that will ensure and support the sustainability of the clinics are needed. Future studies can recruit facilities in other regions and design best practices for the WC.

## Conclusion

The Wellness Clinic is an important initiative, and its implementation shows the political will to address the rising burden of NCDs in Ghana within the primary health system. The WC were positioned to detect and support early diagnosis of NCDs and supported post diagnoses management of NCDs. However, due to poor policy coordination, there was limited knowledge of the structure and function of the WC. This makes it difficult to assess the sustainability of the intervention within the broader health system. To strengthen the WC, there is a need to develop best practices, train staff, and improve coordination at the sub-national level.

## Data Availability

All data generated or analysed during this study are included in this published article.

## Abbreviations

WC: Wellness Clinic
MoH: Ministry of Health
GHS: Ghana Health Service
PHC: Primary Health Care
WHO-PEN: World Health Organization Package of Essential Noncommunicable diseases
